# A qualitative study of the experiences of relatives to brought in dead persons in an emergency department

**DOI:** 10.1111/jan.15384

**Published:** 2022-08-17

**Authors:** Dorthe Gaby Bove, Suzanne Forsyth Herling, Nanna Sørensen, Peter Gjersøe, Helle Timm

**Affiliations:** ^1^ Department of Research Copenhagen University Hospital Hillerød Denmark; ^2^ Centre for Nursing University College Absalon Roskilde Denmark; ^3^ The Neuroscience Centre Copenhagen University Hospital, Rigshospitalet Hillerod Denmark; ^4^ The Emergency Department Copenhagen University Hospital, Nordsjælland Hillerod Denmark; ^5^ The University Hospitals Centre for Health Research, Rigshospitalet Kobenhavn Denmark; ^6^ National Institute of Public Health University of Southern Denmark Odense Denmark; ^7^ University of Faroe Islands Torshavn Denmark

**Keywords:** brought in dead, dead on arrival, deceased, emergency department, emergency nurses, interpretive description, nursing care, prolonged grief disorder, relatives' experiences, unexpected death

## Abstract

**Aims:**

The aim was to explore the experiences of relatives seeing and saying goodbye to brought in dead persons in a Danish emergency department.

**Design:**

This was a qualitative study based on interpretive description methodology.

**Methods:**

Data were collected through semi‐structured individual interviews with relatives (*n* = 11) of brought in dead persons and 30 h of participatory observations of these relatives visiting the emergency department to see and say goodbye to the deceased. Data were collected between February 2019 and December 2020.

**Results:**

Our analyses revealed internal and external chaos as an overarching theme, covering 4 themes and 10 subthemes. The four themes were traumatic events, restricted access, briefly being together again, and final goodbyes and departures.

**Conclusion:**

Emergency departments were highly acute and busy settings that prioritized survival more than the care of deceased people and their relatives. The relatives were, in every way, affected by internal and external chaos; the external chaos in the emergency department reinforced the feeling of internal chaos. It is necessary for managers and nurses in emergency departments to organize and practice care for relatives, whilst bringing in deceased individuals in a less chaotic and more caring manner.

**Impact:**

This study contributes to the knowledge of relatives' experiences regarding brought in dead persons, underpinning the need to care for this subpopulation in emergency departments. Care for relatives of brought in dead individuals has the potential to relieve suffering and prevent diseases, which are core elements of nursing.

## 1 INTRODUCTION

Brought in dead (BID) individuals are those who are unexpectedly found dead or dying outside a hospital, and afterwards taken by ambulance to the nearest emergency department (ED). The responsibility of ED nurses is to care for these individuals and their relatives. As BID persons have died unexpectedly and are subsequently brought to the ED, relatives often visit the ED to say goodbye to the deceased. Although the relatives only stay for a short period in the ED, existing but sparse research indicates that this stay potentially has an impact on their grieving process. Relatives of individuals who suffer an unexpected or traumatic death constitute a vulnerable population with an increased risk of developing prolonged grief disorder and post‐traumatic stress disorder, painful conditions with major human and financial costs. We, therefore, need to improve the care offered to relatives of BID persons during their stay in the ED, as care for the bereaved has the potential to relieve both short‐term and long‐term suffering and pain. Despite the significance of care for relatives, the experiences of relatives of BID persons during their stay at the ED to see and say goodbye to the deceased have not been explored.

### 1.1 Background

EDs are designed and organized to save lives, with ED nurses being educated and trained in acute care and life‐saving skills (Beckstrand et al., 2012). However, in addition to caring for acutely ill patients, caring for BID individuals and their relatives also comprises the responsibility of ED nurses. These nurses arrange and coordinate relatives’ visits to the ED and are present to support them whilst they are with the deceased.

BID persons or individuals dead on arrival (DOA) are synonymous terms used nationally and internationally for a person found unexpectedly dead or dying outside a hospital, or someone who died on the way to the hospital or in the trauma room before being admitted. Only sparse knowledge exists about the number and characteristics of BID persons, which is why we conducted a prior study describing the characteristics and number of BID persons received in a Danish ED. Our results showed that a medium‐sized Danish ED, on average, received one BID individual every day, which made it a frequent occurrence. The majority of the BID persons (80%) were found either dead or dying in their homes, and in 54% of the cases, by a close relative (Bove et al., 2021). On average, each deceased person is known to have four close relatives (Shear et al., 2013).

Care for relatives of a deceased is integral and described as part of palliative care; thus, in a context where death is expected and a relationship between patients, relatives and healthcare professionals is already established prior to death. The environment in an ED is fast‐paced and often somewhat chaotic, with a high patient flow. The ED is equipped with the latest life‐saving technology, and instrumental skills are highly valued and crucial in trained nurses. Several studies have described how ED nurses perceive death as a failure and the ED as an inappropriate place to die (Hogan et al., 2016). A few studies have explored the care needs of the bereaved in an acute setting (Mayer, 2017; Merlevede et al., 2004). However, none have done so in a setting transferrable to modern Danish or European EDs.

Even though the relatives of BID persons only stay for a short period in the ED, existing research on grief indicates that this stay may have an impact on their grieving process; as relatives of individuals who suffer an unexpected or traumatic death constitute a vulnerable population with an increased risk of developing prolonged grief disorder (PGD) (Lobb et al., 2010; Stroebe et al., 2007). PGD differs from natural grief due to its intensity and duration of more than 6 months, being associated with intense emotional pain, depression, anxiety, cancer, cardiac events and reduced quality of life (Djelantik et al., 2020; Lundorff et al., 2017).

The relatives of BID individuals comprise a vulnerable population because the circumstances related to their loss are often associated with some kind of traumatic event (Bove et al., 2021), and the increased risk of PGD (Djelantik et al., 2020; Lundorff et al., 2017) also magnifies the risk of developing post‐traumatic stress syndrome (PTSD) (Kaltman & Bonanno, 2003). Our previous work (Bove et al., 2021) illustrated that relatives (>50%) were often the ones who found the deceased already dead or in a dying state. In other cases, relatives had witnessed or participated in failed resuscitation attempts or were told by the police that their loved one had died by suicide or was killed in an accident. All these traumatic events can cause the bereaved to be in a state of shock when attending the ED. When in shock, some of the instant reactions are rapid information processing and changed perception of time, sharpened senses, absence of emotions and reactions, or overreactions with anger, panic or hysteria (Dyregrov, 2004).

Although it is not possible to generalize the way grief affects individuals, the ‘Dual Process Model of Coping with Bereavement’ (Stroebe & Schut, 1999) offers a theoretical framework for understanding the complexity of grief. The grieving process is perceived in the form of two tracks: loss oriented and restorative. The loss‐oriented track focuses on the internal emotional reactions that the bereaved must cope with due to their loss and the realization that the deceased is irrevocably gone. In the restorative track, the bereaved must cope with their new reality without the deceased and eventually adapt to new behaviours, routines and habits that do not include the deceased. The natural grieving process takes place in a pendulum between these two phases, wherein the loss‐oriented and internal emotional processes are mostly present in the beginning, being gradually replaced by the external and problem‐oriented restorative process. As BID individuals' relatives attend the ED within the first few hours or days after their loss, their grieving process can be expected to be dominated by internal emotional reactions, such as sadness, meaninglessness, despair, denial and anger (Stroebe & Schut, 1999).

Most BID persons are elderly and can therefore be assumed to have older relatives (Bove et al., 2021). Old age is a risk factor for both PTSD (O'Connor, 2010) and PGD (Djelantik et al., 2020; Lundorff et al., 2017). Losing a loved one is a natural part of life, and studies estimate that about 85% of individuals go through a normal and uncomplicated grief process (Bonanno & Kaltman, 1999). Thus, this still leaves 15% of people with complications regarding their loss, which are presumably even more amongst relatives of BID persons when considering the illustrated risk factors.

Therefore, we need to improve the care offered to BID persons' relatives during their visit to the ED, as care for the bereaved has the potential to relieve suffering and pain, subsequently contributing to a reduction in the risk of PGD and PTSD. There is a lack of knowledge regarding the experiences of BID individuals' relatives coming to the ED to see and say goodbye to the deceased. Understanding these experiences will be the first step towards providing improved care for this population.

## 2 THE STUDY

### 2.1 Aim

This study aimed to explore the experiences of relatives seeing and saying goodbye to a BID person in a Danish ED.

### 2.2 Design

The study consisted of qualitative interviews and observations with a design based on the inductive methodology of interpretive description, as described by Thorne. Interpretive description is a qualitative research methodology occupied with solving disciplinary and wicked questions by seeking out the kind of knowledge needed to inform and potentially change practice in a credible way (Thompson et al., 2021; Thorne, 2016).

This study forms part of a larger project with the overall aim of improving the care of BID persons and their relatives and will be followed by a study exploring the nurses' perspectives. The project and its methodological reflections are described in detail in a qualitative study protocol (Bove et al., 2020).

### 2.3 Participants

All participants were recruited from the ED at North Zealand Hospital in Denmark between February 2019 and December 2020.

The eligibility criteria for individual interviews were being an adult relative of a BID person, attending the ED, speaking Danish and being willing to give informed consent to participate in the research study.

Eligible participants were screened by the ED nurse responsible for BID individuals' relatives during their visit to the ED. The sampling strategy was a mix of purposive and convenience sampling. We sought the maximum variation in the cause of death of the deceased, including the age, gender and ethnicity of the relatives. The ED nurses' assessment of whether the relatives had the necessary resources to participate in the research study and the rush at the ED determined whether potential participants were invited. The ED nurses' assessment of the participants' resources was purely based on their subjective assessment and in some cases articulated as cognitive difficulties, general frailty or psychiatric disorders.

Eligible informants for interviews were briefly informed about the project and asked for permission regarding the researcher contacting them within 14–21 days for further information and inclusion, if relevant. Fifteen BID persons' relatives were contacted via telephone and informed about the study. Eleven agreed to participate in individual interviews, with four declining due to a lack of mental strength.

The researcher also made observations of the relatives in the ED. It was not possible to plan the observational situations in advance; therefore, the ED nurses called the researcher when they knew that BID individuals' relatives were arriving at the ED. The researcher presented herself to relatives as both a nurse and researcher. The nurse–researcher accompanied one or two ED nurses, supporting the relatives seeing their deceased. She conducted 30 h of participatory observational study between February 2019 and December 2020. Data from the observations consisted of ‘naive’ field notes (Spradley, 1980).

We recruited participants for both interviews and observations until sufficient data were obtained to answer our research questions. During the last phase of the data collection period, we experienced that what we heard and observed seemed to repeat itself in recognizable patterns, and therefore decided that further data collection would be superfluous (Thorne, 2016).

### 2.4 Data collection

Data analyses from both interviews and observations were conducted using parallel and iterative processes. Our interview and observation guide became progressively focused as our insight and understanding increased during the data collection period. The observations were guided by Spradley's recommendation for participant observation (1980) and interviews with an interview topic guide. The topic guide and detailed description of how participant observation was conducted are described in the study protocol (Bove et al., 2020). All interviews and observations were conducted by the first author, who, in addition to being an experienced nurse, was a proficient researcher.

We were cautious throughout the data collection period that there was a risk of the data gathering being comforting and therapeutic instead of explorative. As described by Thorne, an interviewer with a nursing background may inadvertently step out of the role as a researcher and play the role of a nurse, offering the participants advice, solutions and comfort; this may transform the context from being informative to therapeutic, with the participants becoming patients (Thorne, 2016).

To avoid this, the dilemma was articulated and discussed with participants. In some cases, the researcher and participants agreed to bifurcate the interview so that any clinical questions and advice could be addressed. In other cases, bifurcating the interviews was not possible or was perceived as unethical. In such cases, the interviews were paused for a few minutes and continued with the researcher being aware of her role as a researcher. None of the participants wanted to end the interviews ahead of time.

The interviews had a mean duration of 38 min [range 20–71 min]. All the interviews and field notes were transcribed verbatim by the first author. The transcripts were transferred to the software program NVivo 12 and constituted the unit of analysis. NVivo 12 was used as a tool to organize the data and facilitate a transparent trace throughout the analysis process.

### 2.5 Ethical considerations

Throughout the research, we were aware that the topic of this study was sensitive and that the BID individuals' relatives were in a vulnerable situation. All observations and interviews were planned and conducted with the well‐being and safety of the relatives as the highest priority. Besides being an experienced qualitative researcher, the nurse–researcher had years of clinical experience in palliative care, advanced care planning and grief support. The researcher was affiliated to the ED as a researcher and did not take part in the daily clinical work.

To our knowledge, no relatives have suffered any harm due to their participation in the study, and we are convinced that no participants felt under pressure to participate. There have been no situations in which the involvement of additional professional assistance has been necessary or demanded. Further ethical considerations have been elaborated in the study protocol (Bove et al., 2020).

The study was approved by the Danish Data Protection Agency (VD‐20019‐03) but did not require any further ethical approval according to the Regional Committees on Health Research Ethics in Denmark (https://en.nvk.dk/). The study report was guided by COREQ standards for reporting qualitative research (Tong et al., 2007).

### 2.6 Data analysis

The data were analysed thematically, starting immediately after the first interview/observation, and continued as an iterative process during the data collection period. Information from the observations and individual interviews were considered equally important, being interpreted together. The first author kept an analytic journal during the data collection period. The analytic journal was a document in which reflections, tentative interpretations, things to remember or discuss with research colleagues and new questions or topics to be elaborated were noted. This journal was consulted several times during the analysis phase and subsequent preparation of this paper.

The first step of the analysis was to re‐read the transcripts to become familiar with the data and get a sense of ‘the whole.’ Units of meaning were identified and labelled with broad codes as a first identification of ‘what is this about.’ Afterwards, these broad codes were refined, redefined, split or merged into new groups of categories, asking questions such as ‘what does this mean?’ and ‘so what?’. The categories were constantly compared for differences and similarities and finally condensed into subthemes and themes, illustrated with selected quotes. Finally, the first author relistened to all the interviews and reread all field notes to ensure that the participants' voices could be found in the interpreted results. The findings were presented as themes and subthemes and illustrated with selected quotations. To ensure the anonymity of the participants, their age or relationships with the deceased were omitted from the quotations in some cases.

### 2.7 Rigour

To enhance the credibility and transparency of this study, reflections, methodological considerations and choices were described in the qualitative study protocol (Bove et al., 2020). All phases, from scaffolding the study to analysis and conclusion, were conducted through collaboration and discussions within the research group. In each section, we have described our reflections and actions towards strengthening the credibility of this study (Thorne S. 2016).

## 3 FINDINGS

The 11 interviewed study participants ranged in age from 45 to 80 years and were either spouses (*n* = 7) or sons/daughters (*n* = 4) of the deceased. The ages of the BID persons related to the interviewed participants ranged from 62 to 89 years, with nine of them being male. In one case, the cause of death was suicide, and in 10 other cases, known or unknown disease. In six cases, BID individuals were subjected to cardiopulmonary resuscitation (CPR) either in the ambulance, the ED or at home. The situations and events constituting the observational data were characterized by BID persons aged 29–92 years. The relatives observed exceeded 50 persons and represented all types of family relationships (parents, spouse, siblings, etc.), as well as other close relatives such as friends, acquaintances, neighbours and colleagues. In some cases, the relatives of BID persons were accompanied by their own supportive relatives, such as colleagues or friends.

**FIGURE 1:**
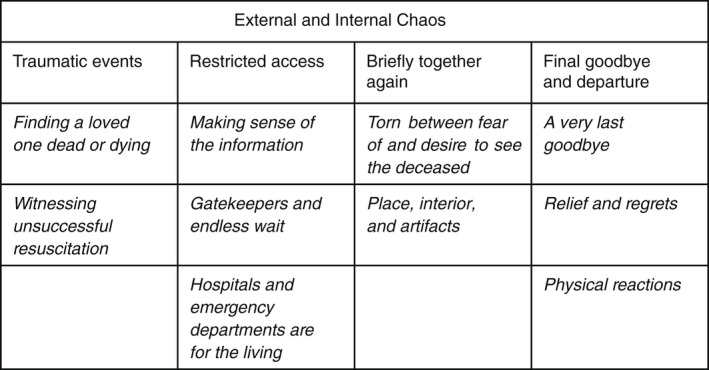
Illustration of themes

Our analyses revealed external and internal chaos as an overarching theme, covering 4 themes and 10 subthemes (Figure 1).

### 3.1 The overarching theme: External and internal chaos

Chaos was identified as an overarching theme that affected all other themes; it was described both internally and externally. The relatives experienced the whole situation as chaotic, affecting their thoughts, emotions, physical conditions and behaviours during the first hours and days, including their visit to the ED: ‘Everything flew by (crying). I cannot even say what I thought of because it was just a huge chaos.’ *(wife of a 74‐year‐old BID individual, ID 10)*.

The ED was perceived as a noisy place, with occupied nurses and doctors rushing to support sick people waiting for help. Patients in beds were driven past the bereaved or placed in the hallway, connected to many technologies and catheters. This whole context was unfamiliar and unsafe, and the relatives described how this external chaos further contributed to their feelings of internal chaos.

### 3.2 Traumatic events

#### 3.2.1 Finding a loved one dead or dying

The relatives described their unpreparedness and how death had come as a shock to them, regardless of whether the deceased was elderly and chronically or even terminally ill. In some cases, the interviewed relatives were those who had found the deceased, either dead or dying, and those who had called for help (911 emergency centre). During the telephonic conversation with the emergency centre staff, the relatives were guided to initiate and continue resuscitation until the ambulance arrived. Several relatives described their failed attempts and frustration, as it did not make any sense to them or it had been physically impossible for them to carry out the CPR:

I am glad he was lying the way he did; he was lying pinned down between the toilet and the shower, with his legs inside the shower. I could not move him, and I do not know if I would have been able to turn him around and bring him to life if he had been lying differently. (wife of a 78‐year‐old BID person, ID 11).

Several relatives described how they relived the situation wherein they found their closed ones dead or dying, and how these flashbacks affected their daily lives in the weeks that followed the death: ‘I get flashbacks that he is lying on the floor in the living room with a notch in his head’ *(son of a 72‐year‐old BID person, ID 4)*.

Dignity or a lack of it was often mentioned by the relatives, because finding a loved one dead or dying was often perceived as an undignified situation for both the deceased and the relatives. The relatives described how they hoped that seeing and saying a last goodbye to the deceased in the ED would replace or erase these last images and experiences, restoring their dignity.

#### 3.2.2 Witnessing unsuccessful resuscitation

Some relatives had witnessed unsuccessful resuscitation, which was a very intense and traumatic experience for them:

I was here in my dressing gown (pointing to the kitchen), and they pulled him out here so he could lie flat and they could start resuscitation. The physician then gave him a syringe directly into his heart. I did not think anything. Occasionally, I looked. I knew that when they pumped, air entered the abdomen, so I knew there was noise along the way. No, I thought nothing. Had I thought clearly, I might have said, ‘Stop, the man is dead, he is not going to a nursing home as a vegetable.’ I might have said so, but it was like it just happened, and I was just watching in a paralyzed state. I just waited and cried, and from time to time, I asked ‘can you revive him?’ (wife of a 78‐year‐old BID person, ID 11).

Being part of or witnessing unsuccessful resuscitation was described as an intense experience involving all senses, especially seeing, smelling and hearing. The experience was further intensified in cases where relatives were left with cleaning and tidying up tasks afterwards.

Being the one initiating the resuscitation was also described as a very unpleasant responsibility and situation, especially in cases where the person had been dead for several hours and was cold:

She had fallen in the kitchen and died. It was my stepfather coming down from the first floor in the morning that found her dead. He started resuscitation and tried to revive her; however, there was nothing to do. He already knew it; he was previously an orderly, but he was told [by 911] that he should. When the ambulance arrived, they immediately declared her dead. (daughter of a 60‐year‐old BID person, ID 7).

Several relatives described how the emergency physician or paramedics subsequently said, ‘How good it was, that we failed resuscitating,’ implying that he or she would never become the same person again. Subsequently, the relatives described how they were puzzled about why resuscitation was initiated in the first place.

### 3.3 Restricted access

#### 3.3.1 Making sense of the information

Death interrupted relatives' everyday lives, causing instant chaos. Several participants had experienced being informed by two uniformed policemen showing up at their doorstep, or being contacted by phone at work or home, either by the police or their relatives.

Several relatives faced difficulty in understanding and processing what was being said. In addition to being shocked, messages were also communicated through unknown concepts and words, such as ‘unexpected death,’ ‘forensic inquest’ or ‘forensic autopsy.’ The relatives had difficulty understanding that the police, as a standard protocol, was involved in ruling out any criminal offences and that this caused a delay, restricting the relatives' access to the deceased; this meant that they were not allowed to touch or be alone with the deceased.

One relative, who had experienced that the deceased was to be autopsied because he was found dead after several days, asked:

How can one be unexpectedly dead when one is 85 years old and suffers from several diseases? At the same time, how can the police state that the cause of death was probably natural, which is the opposite of unexpected and sudden death? (son of an 85‐year‐old BID individual, ID 6).

There were many examples of how relatives were informed by police or ED nurses using references to unknown legislation or rules that, from the relatives' perspectives, were meaningless.

#### 3.3.2 Gatekeepers and endless wait

Several relatives described how they had to struggle to get in contact with the right persons in the ED, and how they felt that some ED nurses acted as gatekeepers and kept them from seeing the deceased. Relatives sat at home waiting, either alone or accompanied by other close ones.

A relative, who had lost her mother, described her experience with the ED nurses through telephone, and how she, at that time, did not understand what she was being told and why she had to wait. It was unclear why the ED nurses had the power to delay or prevent her from seeing her deceased mother, as her mother had already been declared dead at home. However, she did not resist, merely accepting what she was told in the actual situation:

I could not understand why I was made to wait. I was in so much of grief that all I wanted was to see and be with my mother. I was not rational at that moment, but I was accepting what I was told to do. It was all so unrealistic, so I just took one step at a time; since you are so sad, when you are told to wait for half an hour, you wait for half an hour. When nothing happened after half an hour, I just waited a little longer because they [ED nurses] were probably busy. (daughter of a 60‐year‐old BID person, ID 7).

The relatives described their experience of time almost standing still and that everything, except grief and pain, was paused: ‘My father waited and drank beer all day. He drank ten beers in six hours because he could not do anything else. He could not speak. We were stuck in this mourning process and could not get out.’ *(daughter of a 60‐year‐old BID person, ID 7)*.

Telephonic contact with the ED nurses and the waiting time until being allowed to attend the ED to see the deceased was experienced as a very painful situation. The cause of the waiting time was not clear to the relatives; some knew that they had to wait for the police's permission to see the deceased, but in other cases, the reason was unknown or caused by the rush in the ED:

I needed to see him right away, but since he was found dead in his home, the police must be involved to ensure that no crime had taken place. It was difficult to just sit and wait to see him. I was sitting at my mother's house, waiting. I called the ED several times and was transferred from one nurse to another, and told that I just had to be a little patient. I would be allowed to see him, but they could not say at what time. It actually bothered me a lot because I needed to see my father right away. (daughter of an 86‐year‐old BID person, ID 3).

The waiting time was described as awful and amplified by the fact that the whole situation made ‘two minutes feel like several days.’

#### 3.3.3 Hospitals and emergency departments are for the living

The relatives described the ED as a very busy place and an environment occupied with saving lives. The relatives described how they felt that they had disturbed the ED and did not belong there. When they arrived at the ED, they were referred to the waiting room in the emergency room. Several relatives described it as a surreal experience to be placed in an emergency waiting room because they felt that they so clearly stood out in terms of their grief compared to the other persons in the room. At this point in time, the relatives described how they found it rude and provocative that the nurses and physicians laughed and joked in the hallway, and that everyday life continued even though their own lives were chaotic:

Having to wait there, when in shock and grief, was hard. We just stood there and tooted, and everyday life in the ED with casualties and trauma just went by; it was hard. I was not expecting them to participate in our grief, but I wish we were referred to a room where we could be protected from this world. We felt so wrong and vulnerable there [ED waiting room]. (wife of a 60‐year‐old BID individual, ID 5).

Several relatives described the feeling of having disturbed the nurses by both calling and attending the ED. Although not stated directly, they could hear noises in the background and sense the nurses' lack of focus. In contrast, other relatives had not experienced a waiting time and described being met by nurses immediately after arrival, followed and supported during their time with the deceased, and undergoing a subsequent debriefing as the optimal situation for them:

It was important to me, but it was also important for my daughters to have closure: being able to talk about our experiences and how we felt that she [the deceased] was in peace. I did not feel empty‐handed when we left. We had gotten what we came for; the way it was done was just what I could have wished. We were picked up in the ED by two nurses, after which we went to the basement. We walked quietly, it was not like they were rushing anything. It was empathy, real empathy. (husband of a 60‐year‐old BID person, ID 1).

### 3.4 Briefly together again

#### 3.4.1 Torn between fear of and desire to see the deceased

Some relatives were torn between the desire to see the deceased and fear of what they were going to witness. A few relatives did not want to see the deceased, but did so anyway to support their spouse or children. These reservations were both due to past experiences and based on a more generalized fear of seeing a dead person:

The nurses went in first. As they opened the door, my daughter peeked into the room and said ‘argh…I can see her arm.’ After a few minutes, the nurses came out and said, ‘We think it is okay for you to go in and see her now. She looks blue and black, and has not been made presentable in any way because the police did not give us the permission for it.’ Then, they [ED nurses] opened the door and I just went in … I needed to see her. I did not want to see my wife in a closed coffin, and did not know if she was lying in it or what she looked like. Was she at peace? I thought, as a father, I should take responsibility, so I took the girls by hand and went in; the girls responded: ‘Father, you are doing it anyway, though you said you would not.’ I responded: ‘there is nothing to be afraid of; it is our beloved lying in there.’ Thus, we went in and said goodbye. This was good for all of us. (husband of a 60‐year‐old BID individual, ID 1).

Nearly all relatives connected with the deceased by holding hands, touching or kissing their cheeks or mouth. The relatives described how they were surprised by the temperature of the deceased and how they wished they had been prepared for the extreme coldness of the skin. They knew by experience or from movies that a corpse was cold, yet they were surprised to experience how cold it was. Some relatives brought blankets, flowers or drawings to place next to the deceased. It comforted them to provide this last act of care and love, at the same time emphasizing that here lay a person, not a corpse: ‘It was important for me to know that they not only saw him as a dead body, but that they knew who he was as a person.’ *(son of a 72‐year‐old BID person, ID 4)*.

A few relatives appeared to be emotionally unaffected. Most relatives reacted emotionally through quiet or loud crying. Others completely collapsed and had to be physically supported or picked up from the floor. Witnessing the physical changes of the deceased and feeling the coldness of their skin made death and their loss a reality.

Some relatives noticed that the nurses were also emotionally affected by the situation, typically by getting tearful. However, the relatives did not react to or take much notice of this:

At the hospital, we could say goodbye. I felt much better because we now said a dignified goodbye. However, that does not change the fact that my father and I will always have a picture of her at home on the floor; that picture will stick with me forever. (daughter of a 60‐year‐old BID person, ID 7).

Although it was perceived as comforting to see the deceased again, it was not a quick fix that could erase a traumatic experience.

#### 3.4.2 Place, interior and artefacts

The room where the relatives could be with the deceased was situated at the basement of the hospital, about a five‐minute walk from the ED, and nearly all relatives reflected on the specific location:

It was a very dark and gloomy storage room. Now, we have come down into the basement mentally and physically. How far can they [ED nurses] bring you down, that was the feeling I had. (daughter of a 60‐year‐old BID person, ID 7).

Some relatives described the room as ugly, cold, unpersonal and a place deprived of dignity, whilst a few participants did not notice the room at all. The room was small, without windows, and the furniture consisted of two older dining chairs with different designs and two wall lamps. Candlelight and comfortable chairs were missing, being highlighted as factors that could have improved the appearance and cosiness of the room. Some relatives described how they interpreted the location and decor of the room as if they and their deceased were of low priority, comprising a disturbing element in a busy hospital context.

### 3.5 Saying goodbye and departure

#### 3.5.1 A very last goodbye

The relatives described how they felt that saying goodbye was for forever. The bereaved knew that they had to depart from the deceased; however, it was not easy for them to step out of the room and close the door behind them: ‘I was there for a very long time, but eventually, I felt I had to go. I could not let go, but I had to.’ (wife of a 68‐year‐old BID individual, ID 2).

In some cases, saying goodbye was perceived as failing or letting a loved one down:

I think it was hard to leave him again. I think we failed him all along, and it was unfair. It worried me a lot when I was home again. Now, he must lie out there. It was unfair (wife of a 74‐year‐old BID individual, ID 8).

For the relatives, the deceased were still people in need of care, and it was comforting to know that they were still cared for. Some relatives described how important it was to them that the ED nurses used the deceased's name and not just the term ‘deceased.’ By using their name, the deceased's identity was maintained for the relatives, whereas the use of the word ‘deceased’ indicated that the nurses perceived them as a corpse, and not as a person:

I needed to know where he was lying, if he was covered, and if anyone came and looked at him or walked past him, and saw who he was. I still do not know. It does not bother me today, but I wish I knew what happened to him when we left. (wife of a 74‐year‐old BID individual, ID 8).

The relatives stated that it was important for them that the time available with the deceased was not forced, and no one expressed being hurried about leaving the deceased or the ED. During the summer, there were two episodes where the deceased, after being in the room with relatives for quite a long time, needed to be cooled down again. The ED nurses had to make the relatives aware of this, alongside the subsequent need for them to end their visit. The relatives fully understood and accepted this and simply interpreted it as a caring act for the deceased.

A final conversation with the ED nurses after departure from the deceased was not systematically offered to the relatives, as it depended on the time of the day, weekday and rush in the ED. However, when offered, it was highly valued and described as a situation where relatives could finally relax a bit. They did not have many questions for the nurses; instead, they needed to sit down and talk about the deceased, their relationship with the deceased and what led to their death. Sometimes, there were long pauses in the conversation, but the relatives did not want to end the discussion and dragged the time until they were able to mobilize the physical and mental resources needed to get up and leave the ED, and thereby the deceased.

#### 3.5.2 Relief and regrets

In situations where the deceased was either old or suffered from a chronic or terminal illness, the relatives described their relief. A few relatives described how they retrospectively wished that they had never called 911 and that resuscitation attempts had never been initiated:

It is also a relief. We have been under tremendous pressure for the last 2.5 years. I do not know how I got through it. I am not happy she is dead and gone; I certainly am not. I would have preferred to have her here, but when an illness like this, or any other illness for that matter, is so severe, then I think it is okay. I do not blame her; I am just immensely sad that it should end like this. (husband of a 60‐year‐old BID individual, ID 1).

In some cases, relatives' presence at the ED to see the deceased comprised the first time some family members met after several years. Complex and unresolved family conflicts became apparent in such situations and, in some cases, led to guilt and remorse expressed as anger and distrust of other family members:

I was very mad at my oldest brother because he had not responded. I do not understand. I have not told him directly, only indirectly, that it is a shame that the only thing we could do in this situation was to react when he did not pick up the phone. The least we could do when the phone was not answered within an hour was to react. (son of a 72‐year‐old BID person, ID 4).

Several relatives described how they regretted that they had not enjoyed or spent enough time with the deceased before their death. Some relatives of the deceased that were elderly described how they regretted not having discussed their serious illness.

#### 3.5.3 Physical reactions

The relatives had often been through tough times before attending the ED. Some relatives came from far and had either driven a car for several hours or flown all night. Many had had only sporadic sleep and meals in the last 24 h. A few participants consumed large amounts of alcohol and cigarettes.

Sometimes, relatives were offered coffee, juice and snacks during their stay in the ED. They gladly accepted and often asked for more. Foods high in sugar were often chosen, even though relatives did not normally consume sugary things. One relative who usually did not put sugar in her coffee stated, ‘Never mind the coffee, it is the sugar that matters,’ and then poured several teaspoons of sugar into her coffee:

You freeze, and you have nausea, and I had that four days later. You have an inner cold; you are not cold, but you are freezing and nauseous without vomiting, and you have a headache because you are crying all the time. (wife of a 78‐year‐old BID individual, ID 11).

One relative described how she, in addition to the general discomfort, was constantly reminded of the situation due to severe muscle pain in her arms and upper body caused by an intense attempt to save her husband by providing CPR.

## 4 DISCUSSION

The purpose of this study was to describe the experiences of BID individuals' relatives when going to the ED to see and say goodbye to the deceased. Our results illustrated how external and internal chaos was a core theme affecting BID persons' relatives in every way, and how external chaos in the ED reinforced the relatives' feelings of internal chaos.

The experience of internal chaos is somewhat expected within the first hours and days after the loss of a loved one, as previously described in the literature on grief (Clements et al., 2004; Guldin & Guldin, 2019). For the BID individuals' relatives, this internal chaos was amplified by the chaos following an unexpected and unplanned death, traumatic events like ‘finding a loved one dead or dying’ or ‘witnessing unsuccessful resuscitation.’ Restricted access to a BID person and staying in the ED also increased BID relatives' external chaos.

Whilst the internal chaos following death, including an unplanned death followed by traumatic events, has to do with circumstances other than the ED, it seems that ED management and staff could provide care for BID individuals' relatives in a more effective way. The chaos that has to do with ‘restricted access,’ the circumstances of being ‘briefly together again’ and the ‘final goodbye and departure’ are themes that could be acted upon in any ED. Although hospitals are catered for the living, they could, with little effort, also embrace the deceased and their relatives.

### 4.1 Traumatic events

Finding a loved one dead or dying seems to be a traumatic event that cannot be ignored. If people are severely ill, the experience of ‘unexpected’ or ‘unplanned’ death ideally ought to be reduced by palliative care plans or advanced care dialogues. However, our findings illustrated that the BID persons were old and probably suffered from several chronic diseases; death was still unplanned, being perceived as unexpected and shocking to the relatives. Several relatives described how they retrospectively regretted having called for help and having initiated or tacitly witnessed resuscitation attempts, even though they knew it was contrary to the deceased's wish. In addition, health professionals subsequently articulated the failed resuscitation attempts experienced as disrespectful and meaningless. In Denmark, it is only a physician who can declare a person dead unless dead is obvious or expected,—in all other cases, is it an obligation to call for help and start resuscitation. As discussed in a previous paper (Bove et al., 2021), it is debatable whether the death of an old and comorbid person is unexpected and whether resuscitation initiation is appropriate. Our findings illustrate a dilemma between the legal entitlement to carry through resuscitation regardless of age and general state of health, and the subsequent retrospective meaning of having either initiated or silently witnessed resuscitation attempts in a concrete situation. Not starting resuscitation requires that this choice be discussed in advance with a physician, and decisions to opt out of resuscitation be noted in patients' health records. However, Advanced Care Planning (ACP) is rare amongst people not suffering from a terminal illness (Skorstengaard et al., 2020). Older people do frequently attend the ED in their last years of life (Gruneir et al., 2011), and this could be an overlooked opportunity to discuss ACP with older patients and their relatives, regardless of whether the patient suffers from a terminal illness.

### 4.2 Restricted access

The relatives experienced confusion in the use of concepts as well as contradictions in communication with ED nurses. One relative had been denied access to his deceased father because his death was unexpected and required police involvement. It made no sense to him that death could be unexpected when his father was 85 years old and comorbid, or that this unexpected death had to be investigated by the police, who subsequently concluded that the death was natural. This illustrates the need for ED nurses to be aware that BID individuals' relatives are in a chaotic mindset, vulnerable and sensitive. They demand meaningful and honest information and perceive it to be unreliable and unsafe when communication is unclear and distinct. One way to address this could be by limiting the amount of information to a ‘need to know basis,’ handing out written material and offering the relatives a follow‐up conversation with a nurse. Cooper et al. evaluated a grief support program offered to relatives who had lost a loved one to traumatic events. The bereaved were offered support from the ED through in‐person, telephone or email follow‐ups up to 1 year after death. The program was evaluated by the bereaved as meaningful and had a positive influence on their grief processes (Cooper et al., 2019).

### 4.3 Briefly together again

Although the duration of stay in the ED is often short, the intensity and content of the stay may have a long‐standing effect on the relatives' grief reactions (Merlevede et al., 2004). The relatives in our study described how they repeatedly relived the time leading up to their visit to the ED, their communication with the ED nurses, the lack of care or the received care as something of great importance to them in actual and future situations. It can be argued that if the visit to the ED is perceived as an unpleasant and traumatic event, the visit itself can contribute to the sum of events increasing the risk of a difficult or pathological grief process.

Dignity or the lack of dignity was mentioned in relation to the relatives themselves and the BID person. Dignity is a core concept in person‐centred care, and a lack of dignity is associated with psychological stress (Pringle et al., 2015). Persevering dignity for both themselves and the BID person was important to the relatives and, in some cases, violated as the relatives did not feel welcome, being a burden to the busy ED nurses; the relatives also felt vulnerable and exposed during their stay at the ED. They sought protection from the surroundings as their internal chaos and emotional pain were reinforced by the external chaos they experienced in the ED occupied with saving lives. Furthermore, the lack of decoration of the room where the relatives could be with the deceased was perceived as ugly and cold, being located in the basement, which was the lowest‐ranked location in a building. It violated the relatives' feeling of dignity, highlighting that no one had cared enough to place a candlelight or flowers in the room, although it is known that the appearance and location of a room, including artefacts and rituals, are important factors that preserve dignity and relieve suffering (Brereton et al., 2012).

### 4.4 Final goodbye and departure

During their stay in the ED, relatives' senses were sharpened, their sense of reality and time was distorted and their bodies reacted through nausea, headache and/or sugar cravings. Reactions that are known from the literature on disasters and catastrophic psychology can be explained by a ‘fight or flight response,’ with an activation of the sympathetic nervous system and automatic adaptive response, making it possible for the relatives to mobilize their mental resources according to the requirement of the situation (Dyregrov, 2004). Being invited to sit with the ED nurses after being with the deceased and being offered beverages were highly appreciated by the relatives, helping them gather their thoughts and find some peace of mind before leaving the ED. It was not easy for the relatives to leave the ED, as they were aware that it was their last chance ever to be with the deceased. It was important to them that this moment was not rushed, and that they were given the time to prepare themselves for the final goodbye.

### 4.5 Limitations and strengths

The participants were recruited only from a single ED. However, it was never our intent to recruit more than one ED due to the time frame of the study and the ongoing COVID‐19 pandemic. We found that the wide variation in our participants repealed, to some extent, the lack of contextual variation; however, we recommend further research to validate our results by including participants from other EDs.

Thus, a qualitative protocol is not a static document we consider it as a strength that our ‘a priori’ considerations and reflections, including potential limitations and ethics, were described. We did not deviate from the protocol during the study period, except for a slight deviation in the number of observational hours (planned 50 h, conducted 30 h) and lack of variation in the participants' ethnicity (Bove et al., 2020).

## 5 CONCLUSIONS

The BID relatives' experience of seeing and saying goodbye to a BID person in a Danish ED is in every way affected by internal and external chaos, with external chaos in the ED reinforcing their feeling of internal chaos. EDs are highly acute and busy settings that prioritize life‐saving and survival rather than dead people and their relatives. Our findings suggest that the ED nurses could organize and practice care for BID persons' relatives in a less chaotic manner in the ED setting.

## AUTHOR CONTRIBUTIONS

All authors have agreed on the final version and meet at least one of the following criteria (recommended by the ICMJE*): (1) substantial contributions to conception and design, acquisition of data, or analysis and interpretation of data; (2) drafting the article or revising it critically for important intellectual content. *http://www.icmje.org/recommendations/

## FUNDING INFORMATION

This work was supported by The Lundbeck Foundation and a research grant from North Zealand Hospital, Denmark.

## CONFLICT OF INTEREST

The authors declare no conflict of interest.

### PEER REVIEW

The peer review history for this article is available at https://publons.com/publon/10.1111/jan.15384.

## Data Availability

The data that support the findings of this study are available on reasonible request from the corresponding author. The data are not publicly available due to privacy or ethical restrictions. All data are in Danish.
